# Softening the Blow of Social Exclusion: The Responsive Theory of Social Exclusion

**DOI:** 10.3389/fpsyg.2016.01570

**Published:** 2016-10-10

**Authors:** Gili Freedman, Kipling D. Williams, Jennifer S. Beer

**Affiliations:** ^1^Department of Film & Media Studies, Dartmouth CollegeHanover, NH, USA; ^2^Department of Psychology, Purdue UniversityWest Lafayette, IN, USA; ^3^Department of Psychology, University of Texas at AustinAustin, TX, USA

**Keywords:** social exclusion, ostracism, protective orientation, defensive orientation, language

## Abstract

Social exclusion is an interactive process between multiple people, yet previous research has focused almost solely on the negative impacts on targets. What advice is there for people on the other side (i.e., sources) who want to minimize its negative impact and preserve their own reputation? To provide an impetus for research on the interactive nature of exclusion, we propose the Responsive Theory of Social Exclusion. Our theory postulates that targets and sources’ needs are better maintained if sources use clear, explicit verbal communication. We propose that sources have three options: explicit rejection (clearly stating no), ostracism (ignoring), and ambiguous rejection (being unclear). Drawing on psychology, sociology, communications, and business research, we propose that when sources use explicit rejection, targets’ feelings will be less hurt, their needs will be better protected, and sources will experience less backlash and emotional toil than if sources use ambiguous rejection or ostracism. Finally, we propose how the language of rejections may impact both parties.

## Introduction

Imagine you have two friends getting married on the same day in different states: you would be faced with having to accept one invitation but reject the other. Declining a wedding invitation is just one example of social exclusion, which occurs when a perpetrator (i.e., a source) denies a target his or her explicit or implicit social request. For example, an explicit request could be asking a friend to attend one’s wedding whereas an implicit request would be assuming that a friend does not want to sever ties. Social exclusion can occur in either of these situations: a person can say no to a wedding invitation and can choose to end a decade-long friendship. Research has robustly shown that targets of social exclusion suffer a variety of negative effects (e.g., Leary, 1990; Baumeister et al., 2002; Williams, 2007a,b; Slavich et al., 2010; DeWall and Bushman, 2011; Williams and Nida, 2011) but less is known about the sources of rejection (e.g., Poulsen and Kashy, 2012; Legate et al., 2013; Wesselmann et al., 2013; Zadro and Gonsalkorale, 2014; however, for a recent discussion of sources of ostracism, specifically, see Gooley et al., 2015; Grahe, 2015; Legate et al., 2015; Nezlek et al., 2015; Poulsen and Carmon, 2015; Van Tongeren et al., 2015; Wesselmann et al., 2015; Wirth et al., 2015). If one potential goal of research on exclusion is to minimize exclusion’s negative effects, then psychologists need to investigate exclusion from both the target and source’s point of view. In addition to research on targets’ wants and needs, research is needed to understand what sources want and need. Similarly, in addition to research on how targets cope with exclusion, research is needed to understand how sources’ actions lead to the negative effects that targets experience. Investigating these questions will help address whether social exclusion could be perpetrated in a less damaging way.

In cases of unexplored phenomena such as the perspective of rejectors, a theory’s main task is to generate predictions about how people will behave, rather than accounting for a body of existing effects (Gawronski and Bodenhausen, 2015). Therefore, the present article provides a framework to guide future research on social rejection when conceptualized as an interactive process between source and target. Furthermore, the focus of this framework is to understand exclusion in everyday occurrences in which the goal is not to hurt the target. This focus is in stark contrast to existing work, which has examined cases of exclusion in which the goal is to hurt the target. Previous work has often focused on bullies or people who derive power or self-esteem from victimizing others or exclusion resulting from punishment within a close relationship (e.g., Crick, 1995; Olweus, 1995; Prinstein et al., 2001; Zadro et al., 2005; Williams, 2007a; Wesselmann et al., 2013). In fact, one motive for engaging in ostracism is the desire to punish the target (i.e., punitive ostracism; Williams, 1997, 2001). Researchers have also considered other motivations for engaging in ostracism such as trying to preemptively defend themselves from a confrontation, following a prescribed role, and unknowingly ignoring someone who is of a lower status (Williams, 1997, 2001). We propose that a motive that should be more carefully examined is that of wanting to exclude but not wanting to hurt or punish. In other words, sometimes individuals want to end a relationship, prevent one from beginning, or avoid an interaction but do not want to injure the target. In these cases of everyday social exclusion, the exclusion is intentional, but the hurt arising from the exclusion is not. The current framework considers these everyday instances of social exclusion that often arise because it is not always possible or realistic to include others. For example, people may find themselves having to exclude someone when a troublesome roommate wants to renew the lease, an unwanted admirer wants to go on a date, or when two friends get married on the same day. In these everyday instances of exclusion, we propose that sources are not out to harm the target and instead will prefer to exclude in a way that minimizes damage to both themselves and the target.

More specifically, this article proposes a theoretical framework, the Responsive Theory of Exclusion, which differs from existing theories because it takes into account both the sources and targets of social exclusion and draws on research from psychology, sociology, communications, and business. The Responsive Theory of Exclusion proposes that both parties will fare better when sources are responsive to targets’ needs. In general, individuals who display responsiveness are better liked, and interactions with them are more successful than interactions with less responsive individuals (Werner and LatanÉ, 1976; Davis and Perkowitz, 1979). Therefore, we argue that for social exclusion to be a less damaging process for both targets and sources, sources should display a higher level of responsiveness toward targets.

First, we review literature to characterize targets’ needs (meaningful existence, belongingness, self-esteem, and control) and sources’ needs (avoidance of reputation damage, hurt feelings, and emotional effort) during social exclusion. Next, we consider the various forms of social exclusion available to sources. Finally, we analyze the various forms of social exclusion for their potential to fulfill the shared and distinct needs of both targets and sources. Our analysis suggests several hypotheses about how to minimize the damage of social exclusion for both targets and sources. For example, minimizing the negative impact of exclusion is not as simple as being nice. In many cases, targets and sources may be most likely to achieve their needs when sources communicate explicit rejections (as opposed to ambiguous rejection or ostracism) with language that acknowledges both parties in the interaction.

### What Targets Want: Restoration of Self-Esteem, Meaningful Existence, Belongingness, and Control

According to Williams’s (2009) Need-Threat Model, social exclusion threatens four fundamental needs and motivates targets to restore those needs. Many models have characterized the needs that might be related to social exclusion including broader theories on self-regulation (e.g., Self-Determination Theory; Deci and Ryan, 1985) and those more specifically focused on social exclusion. In order to facilitate relation between existing findings on the target and our proposed hypotheses on the source, we have chosen to build on a widely used model focused on social exclusion—Williams’s (2009) Need Threat Model. Our focus on targets’ needs stems from the idea that the crucial point of intervention is through needs, not through consequences. In other words, if sources can reduce the threat to targets’ needs, targets are likely to suffer fewer consequences. Based on a functional account of emotions (Levenson, 1994), it is possible that the threat to one’s needs would precede the emotional and behavioral consequences of social exclusion. However, it is possible that need threat and emotional and behavioral consequences occur simultaneously in response to social exclusion. In either case, it is important for sources to be aware of targets’ needs and to exclude in a way that minimizes need threat.

First, a large body of empirical work has demonstrated that social exclusion impacts four fundamental needs of the target from the Need-Threat Model (Williams, 2009): self-esteem (Leary et al., 1995; Gerber and Wheeler, 2009; Bernstein et al., 2013), meaningful existence (Williams and Sommer, 1997; Williams et al., 2000b; Zadro et al., 2004; Gonsalkorale and Williams, 2007; Young et al., 2009; Garris et al., 2011), belongingness, (Zadro et al., 2004; van Beest and Williams, 2006; DeWall et al., 2008; Romero-Canyas et al., 2010; Hawkley et al., 2011), and control (Warburton et al., 2006; Wesselmann et al., 2010; Schoel et al., 2014). While self-esteem and belongingness are likely to overlap to some degree because self-esteem involves our feelings of belongingness (Leary and Downs, 1995; Leary et al., 1995), self-esteem is also derived from other aspects of the self that are distinct from belongingness, such as competence (Tafarodi and Swann, 2001).

Second, following the exclusion episode, targets are motivated to restore those needs (e.g., Williams et al., 2000a; Williams, 2009; Jamieson et al., 2010). Research suggests that the restoration of these needs is an important avenue for reducing the negative effects of social exclusion. When targets restore one or more of these needs, they experience reduced hurt feelings and engage in less retaliatory aggression (e.g., Warburton et al., 2006; Teng and Chen, 2012).

#### Self-Esteem

Both theoretical and empirical research point to targets’ threatened self-esteem, their motivation to restore it, and the benefits of its restoration. Both the Need-Threat Model (Williams, 2009) and Sociometer Hypothesis (Leary and Downs, 1995; Leary et al., 1995) posit that exclusion undermines self-esteem. According to the Sociometer Hypothesis, self-esteem is a marker of how included or excluded a person feels (Leary and Downs, 1995; Leary et al., 1995). That is, self-esteem is a measure of relational value: how much others value the relationship. By definition, exclusion indicates that a target’s relational value is diminished: the source does not value the target enough to include the target in the requested social interaction. Similarly, the Need-Threat Model posits that social exclusion threatens targets’ self-esteem by indicating that the target is not valued enough to be accepted. Furthermore, the Need-Threat Model also describes social exclusion as impacting self-esteem through the potential ambiguity of the situation (Williams, 2009). For example, when the situation is ambiguous, targets may develop lay theories about the reason for the social exclusion that might make their negative traits and actions more salient.

There is extensive empirical support for the negative effect of exclusion on targets’ self-esteem and their need to restore it following exclusion (for reviews, see Leary, 1990, 2005a; Williams, 2007a). Even in situations in which targets think that the exclusion did not make sense, and they disagree with the action, they still exhibit decreases in self-esteem (Leary and Downs, 1995; Leary et al., 1995). In fact, merely seeing someone look away, instead of directly at the target, can lead to feelings of relational devaluation (Wirth et al., 2010). When targets are unable to restore their level of self-esteem, they show detriments in other areas of their life. People who fail to restore their self-esteem following an exclusion (i.e., those with vulnerable baseline levels of self-esteem) do not benefit from the usual buffering effects of companionship (Teng and Chen, 2012), show decreased ability to engage in self-control (vanDellen et al., 2012), engage in self-blame attributions, and show increased stress reactivity (Ford and Collins, 2010). Impression management can affect targets’ willingness to admit that their self-esteem has been threatened, especially in an experimental context (Bernstein et al., 2013). When targets are not concerned with how others view them, they admit to lower levels of self-esteem. When targets *are* concerned with self-presentation, they do not admit to lower levels of self-esteem, but they show decreases in implicit self-esteem (i.e., self-esteem levels that do not depend on self-report: Bernstein et al., 2013).

After social exclusion, targets attempt to restore their self-esteem. Some research suggests that targets try to restore self-esteem by paying attention to positive social cues. For example, people who have experienced exclusion and feel a threat to their sense of self-esteem prefer to work with others who are displaying Duchenne (i.e., real) smiles vs. non-Duchenne (i.e., fake) smiles (Bernstein et al., 2010). In summary, both theory and empirical research point to the impact of exclusion on self-esteem as well as the motivation to restore self-esteem following exclusion.

#### Meaningful Existence

Targets also experience a threat to and a desire to restore their sense of meaningful existence after exclusion. Exclusion undermines targets’ sense that other people see them and acknowledge their existence (Williams, 2001). When targets are socially excluded, they can feel as though sources do not consider them to be worthy of even basic acknowledgment. For example, recipients of social exclusion experience threats to their sense of meaningful existence whether the interaction occurs in person (Williams and Sommer, 1997), virtually (Williams et al., 2000b), by an inanimate object (Zadro et al., 2004), by in-group members (Garris et al., 2011), or by a hated outgroup (Gonsalkorale and Williams, 2007). Even vicarious exclusion, such as the rejection of one’s political candidate in an election, can trigger feelings of diminished meaningful existence (Young et al., 2009). Finally, the negative effects of social exclusion on meaningful existence are cross-cultural: members of both independent and interdependent cultures experience a diminished sense of meaningful existence following social exclusion^1^ (Garris et al., 2011; see Ren et al., 2013 for evidence that restoring meaningful existence after social exclusion occurs more quickly for people with interdependent self-construals).

The restoration of feelings of meaningful existence has been suggested as an explanation for one of the most damaging consequences of social exclusion: aggression. Targets may attempt to restore their diminished meaningful existence by engaging in attention-seeking behaviors, some of which may be violent. One theory behind school shootings is that the shooters were socially excluded by their peers and sought to regain their sense that others were aware they existed (Williams and Nida, 2011). In summary, the impact of exclusion on meaningful existence is pervasive regardless of whether it occurs in person or in a more distal fashion, and the desire to restore it may be a reason that targets react with aggression.

#### Belongingness

Following social exclusion, targets also attempt to restore their threatened sense of belongingness (e.g., Williams et al., 2000a; Zadro et al., 2004; van Beest and Williams, 2006; Carter-Sowell et al., 2008; DeWall et al., 2008; Knowles et al., 2010; Romero-Canyas et al., 2010; Hawkley et al., 2011; Riva et al., 2014). Exclusion strips away the sense that one belongs to the group or dyad. In fact, the threat to belongingness is often considered the core threat of social exclusion (Smart Richman and Leary, 2009). After experiencing exclusion, targets show an increased desire for belongingness through socially motivated behaviors and perceptions, namely increasing social interactions with others and seeing the world through a lens of social connection.

After exclusion, targets attempt to restore their sense of belongingness by trying to make new friends and ingratiating themselves with others (Maner et al., 2007; Romero-Canyas et al., 2010). For example, people higher in loneliness are more likely to smoke cigarettes than people who are not lonely, but only if smoking is the norm in their locale (DeWall and Pond, 2011). The desire to restore belongingness following social exclusion also impacts attentional processes: targets of social exclusion pay more attention to social cues than people who have not recently experienced social exclusion. For example, targets view others in a more positive light, selectively attend to positive social images, and show a selective memory bias for social information regardless of the valence of the information (Gardner et al., 2000; Maner et al., 2007; DeWall et al., 2009). In summary, social exclusion threatens belongingness, and targets attempt to regain belongingness through ingratiation as well as enhanced attention and memory for social (compared to non-social) information.

#### Control

Finally, in addition to self-esteem, meaningful existence, and belongingness, targets of social exclusion also want to restore their sense of control. Social exclusion may undermine the target’s sense of agency over the situation. Williams’s (2009) Need-Threat Model of ostracism contends that ignoring the target takes away the target’s ability to respond and therefore the target’s sense of control. Wesselmann et al. (2010) argue that the various social exclusion paradigms (e.g., life-alone task, group member rejection tasks) all decrease targets’ level of control.

Targets often attempt to restore control by performing fewer prosocial acts and behaving more aggressively (e.g., Twenge et al., 2001, 2007; Buckley et al., 2004; Warburton et al., 2006; Ayduk et al., 2008; DeWall et al., 2010; Coyne et al., 2011). If targets of social exclusion are given a chance to regain control in another domain, they no longer exhibit aggression (i.e., giving hot sauce to someone who does not like hot sauce: Warburton et al., 2006). With both control and meaningful existence restoration, it may seem paradoxical that targets would engage in aggressive or antisocial behaviors to restore their threatened needs as those behaviors may threaten their other two fundamental needs (belongingness and self-esteem). However, targets are unlikely to behave aggressively to restore threatened needs if they feel that belongingness is still possible (Maner et al., 2007). It is only when belongingness feels out of reach that targets will behave in antisocial ways to restore their other needs (Maner et al., 2007). Therefore, research indicates that social exclusion threatens targets’ sense of control, and targets will go to lengths to restore it.

### What Sources Want: Protective Orientation, Defensive Orientation, and Emotional Ease

Existing theories have not deeply considered the concerns of sources; understanding these concerns and the extent to which they align with or contradict the needs of targets is important for understanding how to mitigate the negative consequences of social exclusion. For example, are the negative consequences of social exclusion intended by the sources? On the contrary, research suggests that sources often want to maintain their protective orientation (i.e., they want to protect targets’ feelings; see Shared Need section), which is an interpersonal dynamic known to operate in a variety of social situations (Goffman, 1967; Folkes, 1982; Ciarocco et al., 2001; Chen et al., 2014). Beyond concern for targets’ hurt feelings, sources are also concerned for their defensive orientation (i.e., their own reputations: how favorably they are perceived by others; Goffman, 1967). Finally, sources are concerned with the emotional difficulty of perpetrating the social exclusion (e.g., Folkes, 1982; Ciarocco et al., 2001) and they are often uncomfortable rejecting even when they want to reject (Joel et al., 2014).

#### Defensive Orientation

Sources are not just concerned with protecting the target, they also want to defend themselves against reputation damage (i.e., maintain their defensive orientation). People are generally motivated to have others see them in a positive light (e.g., Rogers, 1957; Baumeister and Leary, 1995; Srivastava and Beer, 2005), and they try to avoid situations that will damage their reputations (for a review, see Leary and Kowalski, 1990). In fact, an entire subfield of social psychology is devoted to the processes people use to manage their self-presentation (i.e., impression management; Leary and Kowalski, 1990). The context of social exclusion elicits these same reputational concerns. Sources are aware that targets will not look kindly on their decision to exclude and may form negative impressions of them (Folkes, 1982; Baumeister et al., 1993; Besson et al., 1998; Tong and Walther, 2011). A study of unrequited love illustrates sources’ concern about their defensive orientation. When writing about their experiences of excluding an unrequited lover, people express concern with how the target will view them and do not want to appear unkind (Baumeister et al., 1993).

#### Emotional Ease

Sources also want to exclude in a way that does not require exhaustive emotional effort. Sources report that after perpetrating social exclusion, they experience guilt (e.g., Baumeister et al., 1993; Poulsen and Kashy, 2012), an emotion that people try to avoid (Tangney et al., 2007). Social exclusion is a difficult and taxing process for sources: it requires effort, which may need to be sustained over an extended period of time (Williams and Sommer, 1997; Williams et al., 2000a; Ciarocco et al., 2001). The difficulty of social exclusion has been demonstrated through a diminished capacity for self-control and increased negative emotions following perpetration of social exclusion. For example, when people are instructed to ignore someone who wants to talk to them, they show decreased performance in subsequent effortful tasks such as squeezing a handgrip or persisting on impossible puzzles (Ciarocco et al., 2001). The logic of this research is that engaging in exclusion sufficiently taps self-control resources such that there is little left for subsequent difficult tasks. Furthermore, even when the social exclusion is agreed upon ahead of time as a social experiment amongst colleagues, the colleagues report negative emotions and describe the experience of socially excluding each other as unpleasant and difficult (Williams et al., 2000a). Sources exhibit a preference for exerting as little effort as possible when excluding targets. When social exclusion takes place online, sources prefer to ignore social requests or use one-click options. For example, on a dating website, sources prefer to use a button that sends a “no, thanks” message to the target instead of composing more individual or extensive responses (Tong and Walther, 2011). Taken together, although social exclusion can be a necessary fact of life, sources of social exclusion find it to be unpleasant and effortful and try to decrease the amount of emotional effort involved.

### Shared Need: Both Targets and Sources Want to Protect Targets’ Feelings

**FIGURE 1:**
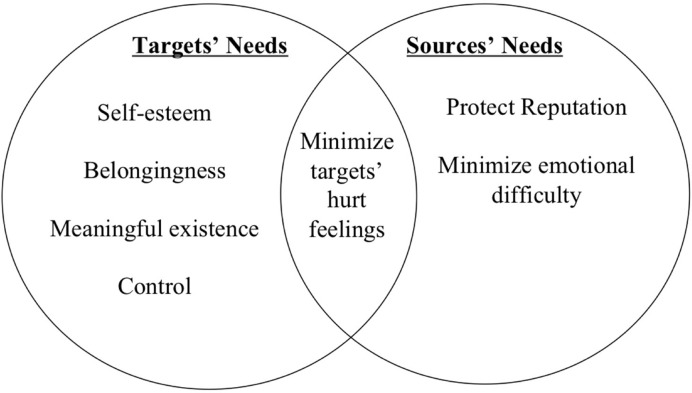
**The shared and distinct needs of targets and sources that are impacted by social exclusion**.

Both targets and sources want to protect the target from hurt feelings. One of the main concerns that sources of social exclusion espouse is their desire to maintain their protective orientation: sources worry about hurting the target’s feelings (Goffman, 1967; Folkes, 1982; Baumeister et al., 1993; Besson et al., 1998; Tong and Walther, 2011). Sources’ concerns about their protective orientations indicate that targets and sources have a shared need: they both want to minimize targets’ hurt feelings (see **Figure 1**). Hurt feelings arise when individuals perceive that others do not value them as members of a relationship (Leary et al., 1998). Furthermore, hurt feelings increase to the degree that people feel devalued (Leary et al., 1998). In fact, one of the main causes of hurt feelings is experiencing ignoring or rejection (Leary et al., 1998; Feeney, 2004), and hurt is considered the hallmark emotion of exclusion (Smart Richman and Leary, 2009). Hurt feelings are associated with long-term consequences including damage to self-esteem and confidence in future interactions as well as lasting negative emotional reactions (Leary et al., 1998).

It may seem paradoxical that a source will exclude a target but claim to not want to hurt their feelings. Why not just include the person? Unfortunately, it is not always possible to include others. For example, people may find themselves having to exclude someone when a troublesome roommate wants to renew the lease, an unwanted admirer wants to go on a date, or when two friends each hold their weddings on the same day. Sources’ concern for targets is illustrated by research on sources rejecting an unrequited lover’s advances and by research on sources’ strategizing to communicate social exclusion. In situations of unrequited love, sources find it unpleasant that their goal to deny the romantic request conflicts with their goal to avoid hurting feelings (Baumeister et al., 1993). Research on communication strategies indicate that sources attempt to use language that they believe will diminish the degree to which targets experience hurt feelings. For example, in one study, participants were asked to report their true reasons for engaging in social exclusion and to report which reasons they would actually provide the target (Folkes, 1982). Out of concern for the targets’ feelings, sources tried to avoid providing reasons that they believed would hurt the target (e.g., stable or uncontrollable aspects such as the targets’ appearance or personality; Folkes, 1982). In summary, just as targets of exclusion do not want to feel hurt, sources of social exclusion often do not want to hurt targets’ feelings.

### The Dyadic Nature of Exclusion: A New Factor for Categorizing Types of Exclusion

**FIGURE 2:**
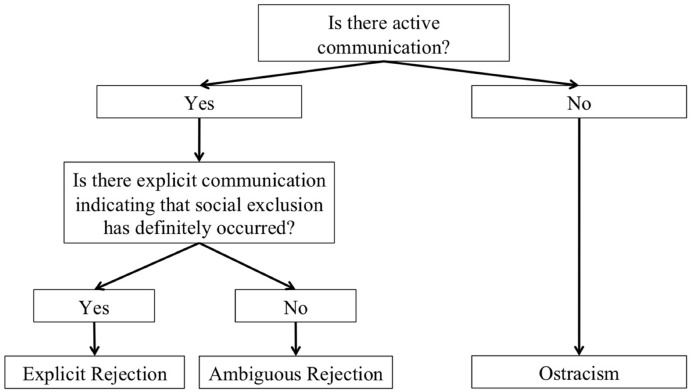
**The different forms of social exclusion described by the Responsive Theory of Social Exclusion: explicit rejection, ambiguous rejection, and ostracism**.

In addition to understanding the needs of both sources and targets, a fundamental understanding of social exclusion requires a taxonomy of the forms social exclusion (see **Figure 2**). What forms of social exclusion are available to sources when they are trying to meet their needs and the needs of targets? Previous research has categorized forms of social exclusion based on a variety of different factors including the degree to which the exclusion was active vs. passive and explicit vs. implicit (Leary, 1990, 2005b; Williams, 1997; Molden et al., 2009). Our taxonomy instead conceptualizes the difference between forms of social exclusion in terms of how inclusive they are to the target and what they require of the source. In other words, how are the target and the source communicating? In order to understand social exclusion as a dyadic process involving both a target and a source, it is paramount to consider the way in which the source communicates with the target, and if the target has an opportunity to communicate with the source. The advantage of our taxonomy is that it allows for future research to evaluate social exclusion not just in terms of the impact on the target but also in terms of the impact on the source and the relationship between target and source. Specifically, we propose three categories of social exclusion that vary in whether the exclusion involves clear, explicit verbal communication: explicit rejection, ambiguous rejection, and ostracism (defined below).

Most previous conceptualizations of social exclusion have focused on either the perspective of the target or the source, which is problematic because it does not allow for research to consider the dyadic effects of social exclusion. For example, the source’s level of activity has been used to categorize types of social exclusion. In the active-passive continuum, ignoring someone is considered passive whereas avoiding someone is considered active. Furthermore, explicitly rejecting and ostracizing are considered to be two of the most active forms (Leary, 1990, 2005b). However, when considering the dyadic nature of social exclusion, the level of activity of one party is not the crux of the issue. Instead, the interaction, that is, the communication between the target and the source is paramount. For example, explicit rejection involves the source communicating with the target and acknowledging the target as part of the interaction. However, ostracism does not allow for any communication, yet both are considered active. For both target and source, the effects of ostracism vs. explicit rejection will likely be different because of the amount of communication involved.

A second approach to categorization has been to consider the extent to which the exclusion is explicit or implicit to the target (e.g., direct verbal communication with the target vs. or indirect/no communication with the target; Molden et al., 2009). This differs from the active-passive categorization because it focuses on whether the target has direct feedback about the social exclusion rather than how active the source has to be. Yet the consideration of the level of explicitness or implicitness of the social exclusion does not paint a complete picture of the social exclusion dynamic. Indirect and no communication are both captured by the implicit category, but it is important to consider the differences between indirect (or ambiguous) exclusion and no communication (i.e., ostracism). That is, social exclusion is not always clearly explicit or clearly implicit which means a third category is needed. Specifically, communication may occur but not in a clear manner. For example, if a source tells a potential romantic partner that he or she is someone the source would want to date, but not now, there is communication but the result is ambiguous for the target. Therefore, it is important to consider not just explicit vs. implicit, but also separately consider times when the exclusion occurs in an ambiguous manner.

### A New Taxonomy: Ostracism, Ambiguous Rejection, and Explicit Rejection

Our taxonomy builds off of the previous research on forms of social exclusion by conceptualizing social exclusion to the degree it includes clear, explicit verbal communication (explicit rejection) or not (ambiguous rejection and ostracism). Ambiguous rejection is distinct from ostracism, that is lack of any communication, because it may involve verbal communication (note that ostracism has sometimes been used to indicate a degree of verbal communication which is distinct from how the term is being used in the current article: Williams, 1997). Ambiguous rejection is distinct from explicit rejection because it contains a mixed response to the request for inclusion.

#### Explicit Rejection

Explicit rejection occurs when a source communicates with the target and states that he or she is denying the target’s social request. The communication may happen in a more or less active manner (e.g., in person, phone call, email, virtual message, text). The distinguishing feature of explicit rejection is that the source’s verbal communication provides a clear answer to the target’s implicit or explicit request for inclusion. For example, someone might say “I’ve had fun talking to you, but I don’t want to go to lunch with you” while another person might respond to an email by saying, “I do not have any interest in spending more time together.” Both cases are examples of explicit rejection because there is verbal communication that makes it clear that inclusion for the particular social request is not going to happen.

#### Ostracism

Within our taxonomy, we define ostracism as a form of social exclusion that occurs when the source ignores and excludes the target and does not provide any indication that the target will receive an answer to the social request (Williams, 2001; Molden et al., 2009). In other words, we use the term ostracism to describe social exclusion that is accomplished without any verbal communication with the target, which is the way it has often been used in the social exclusion literature (e.g., Williams, 2007a). This may occur with little or great effort depending on how likely the source and target are to come in contact with one another notwithstanding the ostracism. Although the origin of the term ostracism is the use of *ostraca* (shards of pottery with names on them) to expel people from ancient Athens (Williams, 2001), for the purposes of contemporary theory, we focus on ostracism as the silent treatment without an announcement of why it is occurring.

#### Ambiguous Rejection

In contrast to ostracism, ambiguous rejection does involve communication with the target. As with explicit rejection, the communication may be more actively or passively delivered. Despite their element of communication, ambiguous rejections do not include clear statements as to whether the social request is denied or accepted. In other words, ambiguous rejections occur when the source provides a mixed message to the target.

Ambiguity may operate at one or more levels such as inconsistent content of the message, a mismatch between verbal and non-verbal cues, and/or a mismatch between the source’s communication and actions. Inconsistent content occurs when the source provides conflicting information within the rejection. For example, the source can ambiguously reject the target’s request to go to lunch by stating, “Yeah that sounds good, let me think about it.” The rejection is unclear because the first part (“Yeah that sounds good”) implies that the answer is “yes,” but the second part (“let me think about it”) implies that the answer may be “no.” A mismatch between verbal and non-verbal cues also fails to send a clear answer. For example, if the source states, “yeah, sure” to the lunch request but is furrowing their eyebrows and looking askance, the true answer becomes unclear. Finally, the source’s words and subsequent actions can also create an ambiguous situation for the target. For example, if the source tells the target, “I can’t this week—how about next week,” but then fails to set a time with the target for the next week, the target is left unsure of the true intent of the suggestion to spend time together. It is important to note that an ambiguous rejection necessitates that the source does intend to reject the target but may use ambiguous communication for a variety of reasons (e.g., lacking confidence to be direct with the target, wanting to let the target down gently, etc.).

### Analyzing The Potential Effect of Forms of Exclusion on Targets’ and Sources’ Needs

Considering both the source and target of social exclusion generates new avenues for thinking about how to mitigate negative consequences. Previous research has asked the question of how targets can mitigate the negative consequences of social rejection and found that targets can restore their damaged needs but sometimes these restorative efforts engender further damage. For example, when targets experience threat to their sense of control or meaningful existence, they sometimes lash out aggressively at sources (Warburton et al., 2006; Williams and Nida, 2011). They can also behave aggressively toward innocent bystanders, which reveals the need to intervene prior to the social exclusion and not just after (Williams and Nida, 2011). The Responsive Theory of Exclusion takes a different approach by asking a different question: How can sources execute social exclusion in manner that can protect needs from the outset? If social exclusion can be executed in a less destructive way, targets may experience fewer threats to their needs and therefore behave more adaptively. In the following sections, we discuss how each form of social exclusion may impact targets and sources’ needs.

### The Responsive Nature of Explicit Rejection Will Best Satisfy Target and Sources’ Needs

If targets and sources share the goal of protecting the targets’ feelings, perhaps the best place to begin thinking about how forms of exclusion impact targets and sources is in the domain of hurt feelings. Previous research indicates that the best way to combat the hurt caused by exclusion is to engage in inclusion (e.g., Tang and Richardson, 2013). Therefore, we predict that explicit rejection will be most likely to preserve targets’ feelings because explicit rejection has more of an element of responsiveness than ambiguous rejection or ostracism. The clear, verbal communication of explicit rejection has the potential to provide the target with a sense of inclusion in the process.

Beyond the shared goal of preserving the targets’ feelings, targets have their own distinct goals: they want to maintain their four fundamental needs (Williams, 2009). We argue that explicit rejection will also be the best choice to maintain targets’ self-esteem, meaningful existence, belongingness, and control. Overall, explicit rejection can buffer targets’ fundamental needs by indicating that the targets belong to both to the world at large and to the dyad even if the source denies a particular social request. Targets’ self-esteem, control, belongingness, and meaningful existence may fare better when they sense that, although the source is excluding them from the desired social request, sources are still going to lengths to include them in direct communication (i.e., providing a positive social cue) rather than ignoring or sending mixed messages. The target can also experience a sense of control over the outcome of the social exclusion when it is delivered as an explicit rejection. The target knows that the exclusion has taken place and can decide on the next step forward. In explicit rejection, the targets can respond and have an active role as the exclusion unfolds (e.g., communicate that it is not a big deal, argue back, etc.).

Not only do we argue that explicit rejection will best achieve the shared need of protecting the targets’ feelings and the four distinct needs of the target, we also argue that explicit rejection will be most likely to satisfy sources’ distinct needs. Specifically, we predict that sources who engage in explicit rejection will be seen by the rejected party in a more positive light and will have to expend less emotional effort. For example, in the business domain, rejected applicants state that they would rather receive explicit and straightforward rejections as opposed to ambiguous or non-existent rejection communications (e.g., Brown, 1993; Waung and Brice, 2000). Furthermore, job applicants have the most negative reactions to companies that do not provide explicit rejections (Brown, 1993; Waung and Brice, 2000). Rejected applicants look more favorably upon a letter that clearly states that they did not receive the job offer than never receiving any letter (Brown, 1993; Waung and Brice, 2000). Although business rejections and social rejections differ in a variety of important ways (e.g., power dynamic, the source’s amount of choice and agency), they do share features that may make some advice from one domain relevant for the other. For example, rejection is not only taxing for sources of social exclusion, it is also taxing in the business world (e.g., Grunberg et al., 2006). In fact, managers who fire employees experience a range of health problems because of their emotional exhaustion (Grunberg et al., 2006). Therefore, we hypothesize that explicit social rejections, like explicit business rejections, will damage reputation less than the other forms of social exclusion because targets appreciate a straightforward response.

In terms of emotional effort, if social exclusion were compared to entering a pool of cold water, explicit rejection would be the quick cannonball into the water. It can be difficult to jump in the cold water because what is coming will be unpleasant, but by doing it quickly, the jumper avoids prolonged agony. In other words, we hypothesize that explicit rejection will be the easiest in terms of emotional toil because the upfront investment in crafting a response and facing the target is actually less effortful than prolonged mixed messages or silence. However, it is important to note that explicit rejections do require sources to choose their words carefully.

### Ostracism Denies Targets and Sources’ Needs Through A Lack of Responsiveness

How might ostracism and ambiguous rejection fare in comparison to our proposed benefits of explicit rejection? We predict that both ostracism and ambiguous rejection will thwart targets and sources’ shared need of protecting the targets’ feelings as well as their respective individual needs. It may be that ostracism has the worst consequences, as there is no element of responsiveness; ambiguous rejections at least include some verbal acknowledgment (albeit confusing) of the target.

#### Ostracism Undermines Target’s Needs

If findings from romantic relationships can be extended to everyday occurrences of social rejection, then ostracism may be the worst choice for exclusion if sources want to minimize hurt feelings and make future interactions possible. Specifically, ostracizing a romantic partner during conflict is highly damaging to relationship longevity and is associated with high levels of distress (Rusbult et al., 1986a,b; Gottman and Krokoff, 1989). Furthermore, episodes of ostracism will likely threaten all four of targets’ fundamental needs because when sources use ostracism, they actively stave off inclusion attempts.

For example, ostracism threatens self-esteem because it signals to targets that they are undesirable (Williams, 2001). The connection of ostracism to negative feelings about the self may stem from the evolutionary past: groups of human and non-human animals used ostracism as a method of dealing with deviant members (Williams, 2001; MacDonald and Leary, 2005; Kerr and Levine, 2008; Wesselmann et al., 2012b). In other words, ostracism has long been associated with the negative actions of group members and receiving ostracism may indicate to members that they have erred, decreasing their self-esteem.

In terms of belongingness, targets may be unable to perceive themselves as part of the dyad when the source is ignoring them. On a larger scale, people who are ostracized feel that they are pushed to the outside of the social group and are no longer able to feel that they are a part of the group (Leary et al., 2003). Ostracism is an extreme method of severing belongingness because it not only excludes the target from the social request; it also excludes the target from social interaction with the source and implies that any future interactions with the sources are unlikely.

A lack of acknowledgment by others can make targets feel as though they are invisible or dead, as if their life has no meaning. Merely having strangers avoid eye contact can threaten the sense of meaningful existence (Wesselmann et al., 2012a). Not only can ostracism feel like one’s existence is being stripped away, ostracism is often equated with death. In some societies it is used as the most severe form of punishment (Gruter and Masters, 1986; Case and Williams, 2004), and James (1890, p. 293) famously described being ignored as being “cut dead.”

Finally, ostracism is threatening to the target’s sense of control because targets are not able to respond to the exclusion. With explicit rejection, targets have the option of responding to the exclusion, but ostracism prevents that option. Therefore, the targets experience diminished control in an already negative situation. Tellingly, when targets of ostracism have their sense of control restored in a compensatory domain, they experience fewer negative effects of exclusion (Warburton et al., 2006; Wesselmann et al., 2010). Control is clearly an important aspect of the target’s experience, and ostracism only serves to undermine that aspect.

#### Ostracism May Be Costly for Sources

In terms of sources’ reputations, targets state that the worst rejection is the one that is never conveyed (e.g., Brown, 1993). If a person takes the time to apply for a job or ask for a date, not responding to the request is a breach of the norm of reciprocity (Cialdini and Goldstein, 2004). When sources violate social norms, their reputations are in a precarious position. Social norm violation is associated with a myriad of negative consequences ranging from non-verbal cues of hostility (Chekroun and Brauer, 2004, as cited in Brauer and Chekroun, 2005) to exclusion from a social group (Schachter, 1951). Therefore, we hypothesize that the norm of reciprocity will make ostracism (i.e., not reciprocating any form of communication) a dangerous choice for sources who want to maintain a good reputation.

Ostracism may often also require exhaustive effort: ostracism is the painstakingly slow climb down the pool ladder. Ostracism is ongoing and continuous and requires continuous monitoring (Williams et al., 2000a). Therefore, although there has not been research comparing the relative effort of ostracism and explicit rejection, we predict that ostracism will require more effort due to the time course and need for continuous monitoring. Research involving instructed or recalled ostracism has indicated that ignoring someone or giving the silent treatment requires a sustained effort and depletes mental resources (Williams and Sommer, 1997; Williams et al., 2000a; Ciarocco et al., 2001; Sommer et al., 2001; Legate et al., 2013; Sommer and Yoon, 2013). One issue with instructed ostracism studies is that the negative feelings associated with ostracizing could be due to diminished control and autonomy (as predicted by SDT; Deci and Ryan, 1985). However, when autonomy is removed from the equation by comparing instructed inclusion to instructed ostracism, ostracism is still associated with increased negative affect, and ostracizers try to regain their sense of belongingness (Legate et al., 2013, 2015). Ostracism, though it seems passive on the surface, requires violating the highly ingrained social norms of attending, acknowledging, and responding to a person (Williams, 2007a). In this way, even ignoring email contact from a person that one is never likely to physically run into (such as someone on a dating website), does involve a degree of effort. Therefore, we predict that ostracism will be the most difficult form of social exclusion from the point of view of emotional effort. It is possible that when sources *want* to hurt or punish a target that ostracism may be the preferred method (e.g., Williams, 2001; Wesselmann et al., 2013, 2014, 2015; Nezlek et al., 2015). In fact, recent research reveals that when sources want to ostracize because the target has threatened to ostracize the source (i.e., defensive ostracism), the sources feel less guilt than those who ostracize due to social demand (Gooley et al., 2015). However, the present theory is concerned with everyday instances of ostracism, not punitive ostracism, defensive ostracism, or bullying.

### Ambiguous Rejections Can Cause Confusion for Targets and Are Costly for Sources

Like ostracism, ambiguous rejection may also cause more problems for targets and sources than explicit rejection. As mentioned earlier, sources may choose ambiguous rejection for a variety of reasons including the belief that this approach lets the target down gently. There is still verbal communication between the two parties but the social request is never actually accepted. The potential problem with the idea of ambiguous rejection as a gentle rejection is that the target may not understand it is a rejection at all or wonder why the source is not being direct, leading to further problems. We predict that the inclusive but misleading interaction characteristics of ambiguous rejections will hurt targets because they will feel betrayed when they finally understand the sources’ actions. Additionally, delaying the realization of the rejection is likely to be costly for sources’ reputation and their emotional effort.

For example, ambiguous rejections may cause hurt feelings and reduced self-esteem for targets. Ambiguous rejections may be particularly hurtful because they can initially convey the message that the target has the possibility of being included, yet it is eventually revealed in the end that the target was in fact rejected from the start. The sense that the source may have led the target on could elicit a sense of betrayal in the target. Betrayal is one of the main elicitors of hurt feelings (Leary et al., 1998), and therefore ambiguous rejection may be problematic for protecting targets’ feelings. In addition to hurt feelings arising from a sense of betrayal, ambiguous rejections may also increase targets’ hurt feelings and lower their self-esteem because targets may perceive that sources did not care enough to provide an explicit rejection. Targets may feel that with explicit rejections, sources have to invest time and emotion. Yet with an ambiguous rejection, targets may perceive sources as taking the easy way out. Targets’ self-esteem may suffer if they sense that sources do not value them enough to make the emotional investment of explicitly engaging with them.

Ambiguous rejections are also likely to undermine targets’ sense of control because they place targets in a confusing situation. Targets’ confusion about the ambiguous rejection can range from uncertainty about whether the rejection even occurred (e.g., she had a weird tone of voice when she said, “okay”—was that a yes or a no?) to uncertainty about the details of the rejection (e.g., was it long-term or short-term: did she say no to lunch just this week or in general?). When targets of social rejection receive ambiguous, confusing messages, they may experience a diminished sense of control because they do not know how to respond. For example, if a Taylor asks Jamie on a date and Jamie responds ambiguously, how should Taylor respond? If the ambiguity signaled an acceptance, the appropriate response would be an expression of happiness. However, if the ambiguity signaled a rejection, expressing happiness would be socially inappropriate. The confusion created by ambiguous rejections leaves the target in limbo or powerless to respond without great risk. Finally, targets may try to resolve their confusion by ruminating about the interaction, which prevents them from having any control over moving forward. If the rejection had been explicit, the target would at least know what kind of responses would help them restore their sense of control (e.g., coping strategies). In summary, with ambiguous rejections, targets are left uncertain about how to act and therefore experience diminished control over the interaction and over their own coping.

Ambiguous rejections are not only confusing and hurtful to targets they can also cause damage to sources’ reputations and create an emotionally difficult situation. In a study of unrequited love (Baumeister et al., 1993), both parties viewed indirect messages as undesirable. Those seeking love thought most poorly of those spurning love when an ambiguous rejection was involved. Those spurning love were quick to assert that they were clear and forthright in their rejections (Baumeister et al., 1993). The authors raised the possibility that people may feel less guilty about warding off an undesired admirer to the extent they can refute accusations of giving someone false hope about a romantic connection (Baumeister et al., 1993). What does that mean for the effect of ambiguous rejection on the source’s reputation? It may mean that ambiguous rejections cause the source to appear capricious and inconsistent. Both of these traits are generally undesirable and associated with seeming dishonest (e.g., Zuckerman et al., 1982; Heinrich and Borkenau, 1998; Weisbuch et al., 2010). As a result, the target may view the source’s actions as malicious because they put the target in an uncertain and potentially hopeful situation before destroying those hopes. Therefore, we hypothesize that ambiguous rejection will most likely be harmful to the source’s reputation.

To return to the pool analogy for emotional difficulty, ambiguous rejection is getting part of the way in, pausing, coming back out a little, and repeating this process until finally immersed. The uncertainty of ambiguous rejection means that sources may have to continually reassert their positions until the target finally understands what is happening. Sources may avoid the cold shock of explicitly rejecting the target, but they are trading that cold shock for multiple (perhaps smaller) cold shocks. The situation cannot truly end until both the source and the target are on the same page, or at least have reached some form of understanding. Until they reach that point, the source has to invest emotional energy into sending mixed messages, and therefore an ambiguous rejection has the potential to require more sustained emotional effort than explicit rejection.

### Summary: The Main Tenets of The Responsive Theory of Exclusion

Our review of the literature suggests a new framework for developing hypotheses about exclusion when both the perspectives of the source and target are taken into account. We predict that explicit rejections will be the best way to achieve the shared and distinct needs of sources and targets when compared to ambiguous rejections and ostracism. Unlike ostracism or ambiguous rejection, explicit rejection involves an element of responsiveness for targets because a clear dialog is occurring. With ambiguous rejection or ostracism, the dialog is either confusing or non-existent. Specifically, we hypothesize that explicit rejections will cause the least amount of damage to targets’ feelings, targets’ four fundamental needs (self-esteem, meaningful existence, belongingness, or control) and sources’ reputations. Furthermore, we predict that explicit rejection will involve the least amount of emotional difficulty from sources.

### Future Directions: Individual Differences, Boundary Conditions, and Conceptual Parallels

The Responsive Theory of Social Exclusion provides a beginning framework to help shape future research on the unexplored perspective of the source and the dyadic nature of social rejection. As such, it focuses on general hypotheses that will form the building blocks of initial research. A future step will be to examine individual differences and boundary conditions. For example, how do sources’ beliefs about social exclusion impact their decisions? What individual differences will influence which form of social exclusion will be the least damaging? What is the ideal language to use in an explicit rejection? After research uncovers the main effects of the different forms of social exclusion on both targets and sources, psychological science can begin to explore how social exclusion operates within the confines of different individual and dyadic differences.

#### Individual Differences

Although our theory provides an overarching view of how different forms of exclusion may impact targets and sources, individual differences may also affect the dynamic. One important set of individual differences to consider are those that impact dyads. For instance, attachment styles can shape relationships as well as interpersonal interactions (Hazan and Shaver, 1987). Within the domain of social exclusion, an avoidantly attached person may respond differently to explicit rejection than an anxiously attached person. Avoidant individuals prefer to maintain distance from others and are not comfortable with emotional closeness (Hazan and Shaver, 1987). Therefore, as both targets and sources, they may actually prefer ostracism vs. explicit rejection: they may not have the same need to sense inclusion as people who are not avoidant.

Similarly, the predictions of the Responsive Theory of Social Exclusion may be bounded by the targets and sources’ levels of rejection sensitivity. People who are rejection sensitive expect and worry about being rejected, and they have exaggerated reactions when they are rejected (Downey and Feldman, 1996). We predict that explicit rejection may be particularly important for individuals who have high levels of sensitivity, as they may be likely to experience even greater negative consequences in the case of ambiguous rejection or ostracism. Although distinct from rejection sensitivity, research on rejection and neuroticism provides evidence that ambiguous rejections may be especially difficult for people with higher levels of neuroticism. Specifically, people with high levels of neuroticism feel an even greater sense of diminished control, compared to people with low levels of neuroticism, when they are unsure whether or not they have been rejected (Boyes and French, 2009). However, the benefits of explicit rejection may be somewhat lost on people who are very low in rejection sensitivity. If someone is very unconcerned about rejection, then its particular form may have less of an effect on that person’s sense of self and mental health. Therefore, it is possible that the degree to which ostracism and ambiguous rejection harm targets may vary based on the targets’ levels of rejection sensitivity.

Furthermore, the ways that targets and sources interact may differ based on the ages of the two parties. For example, the Responsive Theory of Social Exclusion assumes that individuals have both a defensive orientation and a protective orientation, but children who are still learning about how others think and feel may be less concerned with others’ feelings, especially when they are in a more egocentric stage (Elkind, 1967). Even children as young as four and years of age show responsiveness and a concern toward others (Kochanska and Murray, 2000). On the other hand, younger children may at times be more attuned to the feelings of others than adolescents: concern about the self and self-presentation increases with adolescence, which could potentially leave less cognitive space for engaging in a protective orientation (Elkind, 1967). Therefore, it will be important for future research to consider how sources think about social exclusion across the lifespan.

### Boundary Conditions: Explicit Rejection Content and Structure, Relationship Characteristics, and Cultural Influences

#### Explicit Rejection Content and Structure

As stated above, we hypothesize that explicit rejection has the most potential to fulfill both the shared and distinct needs of targets and sources. However, not all explicit rejections are created equal. What content makes some explicit rejections better able to satisfy both parties’ needs? Previous research on business rejections, interpersonal communications, and interpersonal interactions provides a starting point for considering the content of social rejections. Business rejection research suggests incorporating positive regard for the target as well as alternatives to the denied request. In communication research, Politeness Theory (Brown and Levinson, 1987) cautions against the use of apologies. Finally, research on reciprocity in interpersonal interactions provides a foundation for considering the length of the rejection with respect to the rejection situation.

##### Positive regard and alternatives: the importance of feasibility and sincerity

In business rejections, people dislike the company less and are more willing to go back and purchase the company’s goods if the rejection includes alternatives and positive regard (Aamodt and Peggans, 1988; Feinberg et al., 1996; Locker, 1999). In the context of job applicants, alternatives are communicated as possibilities for future interactions with the company (e.g., we will keep your application on file), and positive regard is communicated as appreciation for the applicant (e.g., it was great to meet you at the interview; Aamodt and Peggans, 1988; Feinberg et al., 1996; Locker, 1999). Following this rationale, in social rejections, alternatives should communicate possibilities for future interactions with the source (or sources), and positive regard should communicate that the source values the target in some way.

However, business rejections and social rejections occur in contexts that vary in a number of ways, and the question becomes whether these two strategies will have positive effects in both domains. There are two key aspects that we propose are necessary for alternatives and positive regard to be successful in social rejection: feasibility and sincerity. If sources can provide feasible and sincere statements of an alternative and positive regard, then they should be able to maintain the targets’ four needs and maintain a successful protective orientation by creating an emotional buffer. Furthermore, sources should be able to satisfy defensive orientation because positive regard and alternatives should help their reputation and ease the emotional burden.

*Feasible and sincere alternatives*. When the source presents the target with a possibility of a future interaction (i.e., an alternative), it highlights the limited scope of the denied social acceptance. Additionally, the target has control over whether to agree to the possibility. However, these benefits will only be realized if the possibility of future interaction is feasible and sincere. For example, consider a situation in which a friend asks to join your weekly lunch group with some of your colleagues. You may have to reject the friend’s request for inclusion because you know that group does not want another person added to the lunch. Yet you can offer to personally go to lunch with your friend on another day. That type of alternative provides a real possibility of future interaction just not in the form requested and gives the friend the option to turn down that request, which restores the balance of control. In contrast, if the source of a romantic dissolution offers platonic friendship to the former partner (“I hope we can still be friends”), the offer is unlikely to reap benefits. If the offer is not sincere, then the source may earn a reputation for being patronizing. Even if the offer is sincere, if it is not feasible for the target, the source may be viewed as insensitive, and the target may further lose a sense of control and self-esteem because the ex-partner is capable of being friends while the target falls short of this ability.

*Feasible and sincere positive regard*. Similarly, if sources provide positive regard, that positive regard will only be helpful to the extent that the target believes it to be true (sincerity) and thinks that it is likely given the way the rejection occurred (feasibility). For example, a sincere and feasible form of regard would be stating that the target has positive personality characteristics (e.g., you are a kind, giving person) and leaving out the stereotypical, *however*, statement (e.g., you’re great, but…). In contrast, a statement about the source’s positive feelings toward the target (“I like you”) may be received as less sincere because it might be more challenging for targets to believe that a source would reject someone for whom they have positive feelings. In other words, when the target tries to understand the reasoning behind the rejection, the target may more readily believe that the sources’ feelings changed rather than something inherent to the target changed (i.e., the source may not like the target as much, but the target’s sense of humor did not change). However, future research should test what types of statements targets find the most sincere and feasible. Taken together, we propose that a social rejection will be successful in minimizing damage to both the source and target if it provides a sincere and feasible possibility for future interaction or positive regard. Future research is needed but it is possible that offers for future interactions or statements of regard that are insincere and/or not feasible may be just as damaging as not providing either.

##### Apologies

In contrast to lay intuitions, Politeness Theory (Brown and Levinson, 1987) and research on amelioration after social transgressions raise the possibility that apologies may be detrimental for both sources and targets of social rejections. For example, the principles of Politeness Theory suggest that apologies are likely to threaten a target’s sense of control. People’s responses in social interactions are constrained by social norms (Brown and Levinson, 1987). When targets receive an apology, their set of possible responses becomes limited by norms governing apologies. That is, the normative response to hearing an apology is to express forgiveness (e.g., “that’s okay”). Apologies therefore have the potential to diminish the target’s sense of control by pressuring them to express forgiveness for the rejection before they may actually feel a sense of forgiveness toward the source.

The negative effects of apologies for sources have been seen in the research on social transgressions. Social transgressions occur when someone violates a social norm and harms another person whether intentionally or unintentionally (e.g., accidentally deleting a person’s data by knocking something over or intentionally missing a group deadline and causing a coworker to lose a chance at promotion). In the domain of social transgressions, apologizing is often cited as an ameliorative strategy (e.g., Darby and Schlenker, 1982; Ohbuchi et al., 1989; Hodgins and Liebeskind, 2003; Eaton and Struthers, 2006), but there is an important caveat: apologizing after an intentional transgression makes forgiveness *less* likely (Struthers et al., 2008). The attribution caveat is important because social rejections differ from social transgressions in that social rejections may be more likely to be seen as intentional on the part of the source. For example, if someone wakes up late and misses an important meeting causing distress to coworkers, it can easily be seen as unintentional. However, if someone declines to allow a coworker to join a lunch group, it is harder to see that as unintentional. Since apologies can decrease the target’s sense of control (i.e., based on Politeness Theory) and social rejections may tend to seem intentional on the part of the source, we predict that apologies will backfire when sources use them in social rejections. In other words, contrary to what may seem like common sense, we predict that using apologies within a rejection will decrease a target’s sense of control and decrease the likelihood of later forgiveness for the source.

##### Length

The length of the communication is also an important feature of understanding the consequences of language (Tausczik and Pennebaker, 2010). Length can indicate a variety of things about both the communicator and the communication. For example, a speaker who is talking a lot (i.e., using many words) may just be indicating that he or she is talkative (Tausczik and Pennebaker, 2010). However, more words are also associated with deception (Hancock et al., 2008). If someone is talking too much and providing a lot of detail in response to a simple question, the answer may begin to appear less and less honest (Hancock et al., 2008).

How does length relate to social rejections? We predict that length is an important aspect to consider within social rejections because, even with perfect content, a rejection that is too short or too long may damage both targets’ and sources’ needs. Research on responsiveness in communications support the idea that rejection length may influence the target’s fundamental needs as well as the way the target perceives the source (Davis and Perkowitz, 1979). For example, consider two rejections that have similar content but the first is one sentence and the second is an entire page. It is possible that the target will interpret and respond to those rejections in different ways despite the similarity in content.

For example, rejections that are too short may thwart achievement of both targets and sources’ needs. In terms of targets’ needs, shorter rejections may make the target feel as though the source is brushing them off, which can cause damage to belongingness. Shorter rejections also provide less information to the targets, which denies the target the control of being able to fix the current problem or avoid it in a future situation. Furthermore, targets may feel a lack of meaningful existence and threatened self-esteem if they receive a response that is shorter than they would expect. In terms of sources’ defensive orientation, targets may view sources as callous and uncaring if the rejection seems terse in comparison to the denied request.

However, rejections that are too long may also cause problems for both parties. If a source provides an overly lengthy rejection, it might suggest that the source believes the target will be devastated. In this case, the source has taken away the target’s ability to control the emotional stakes of the social request. For example, if a lunch invitation is communicated in a one-line email, then a lengthy email detailing all of the reasons for rejecting the request will serve to define the exclusion as having much more extreme consequences than originally communicated. A short rejection may earn the source a reputation as a callous person who does not care about the target’s feelings, but a long rejection may earn the source a reputation as a condescending or overbearing person.

We hypothesize two potential guidelines for determining the ideal length for a rejection: expressing sincere thoughts about the rejections using either the length of the social request or the degree of threat the target will experience as a baseline for deciding on length. If sources use the length of the social request as a starting point for the length of the rejection, they may be better able to achieve each party’s goals. For example, if a target sends a one-line email asking a friend to join the friend’s group lunch, the friend should send the target a similar response in both length and format (e.g., a one or two line email). When sources use rejections that have similar lengths to the social request, they show responsiveness and attentiveness to the target. When people are responsive in a conversation, the conversation is more predictable and balances the amount of control each conversant has (Davis and Perkowitz, 1979). When one person is not responsive to another in a conversation, the lack of responsiveness can make the other person feel as though the conversation is not truly taking place (Davis and Perkowitz, 1979). In other words, a lack of responsiveness can feel as though one is invisible and not worth the courtesy of the expected response (i.e., can damage meaningful existence).

What guidelines can sources follow if there is no direct social request? For example, how can a source end a friendship with a troublesome friend? Would a one-line message be equally well received as a page-long message? We predict that if there is no social request to use as a baseline, sources may be able to compose rejections that are commensurate to the threat the target experiences. A threat of greater magnitude (e.g., ending a 10-year friendship) may require more of an explanation and therefore a longer rejection, than a lesser threat (e.g., ending a 10-day friendship).

#### Relationship Characteristics

On the dyadic level, the length and type of relationship between the target and source may impact how each form of social exclusion plays out. For example, on an online dating website, is it more appropriate to ignore an undesirable person’s message or to tell the person that the advances are unwanted? Does the situation change if the two people had already exchanged multiple messages? In the case of the former, it might be more appropriate to ignore the message because the person is a stranger. In the case of the latter, it might be more appropriate to explicitly reject the person because there is now some level of a relationship between the two people: they know each other a little (or a lot, depending on the messages). Interestingly, targets and sources may not agree on the best method in the stranger situation. For example, targets may want an explicit rejection to receive closure and control over the situation. However, sources may be reluctant to provide an explicit rejection because they fear backlash. In the non-stranger situation, backlash may be less likely because there is a cordial dynamic already in place. As such, future research should address how individual and dyadic differences such as attachment styles, rejection sensitivity, and relationship variables may impact the effect of various forms of exclusion on targets and sources’ needs.

#### The Role of Culture

Beyond the individual and the dyad, it is also important to consider how culture may impact the interpersonal nature of social exclusion. One interesting line of research considers how economic subcultures impact perceptions of social exclusion among farmers and herders in Turkey (Uskul and Over, 2014). The economic subcultures of herders and farmers are quite distinct: herders are highly dependent on strangers for their economic livelihood whereas farmers are not. The differences in reliance on strangers play out in situations of social exclusion not related to economic exchange: herders feel more threatened by strangers’ acts of exclusion than farmers and also react with greater willingness to engage in affiliative behaviors after exclusion. In contrast, farmers are more likely to behave aggressively or avoidantly (Uskul and Over, 2014). In terms of broader cultural differences, there is some evidence that people with interdependent self-construals recover more quickly from acts of social exclusion, perhaps because they are more able to think of their other social connections and therefore bolster their threatened belongingness (Ren et al., 2013).

However, the impact of cultures is less clear when it comes to preference for and consequences of different types of social exclusion. For instance, it is possible that cultures that value interdependence may prefer an ambiguous rejection because it may be seen as the least confrontational strategy. Explicit rejections may fail to prevent hurt feelings in interdependent cultures because both targets and sources may perceive them as too direct and potentially offensive. Future research should consider not just how people of different cultures react to social exclusion but how they engage in it.

## Conclusion

If psychological science is to provide advice to people in the unenviable position of having to socially exclude someone, then research needs to tackle the question of what options are available to sources and whether some options are better than others. In contrast to previous discussions, which have mostly focused on targets, The Responsive Theory of Social Exclusion provides a starting point to study the interactive nature of social exclusion. Targets and sources both have needs that can either be threatened or maintained through different forms of social exclusion. By considering each form of social exclusion and how it may impact both targets and sources, psychological research can begin to provide scientific guidelines for sources about how to engage in social exclusion while minimizing negative consequences.

Our review suggests that sources may be able to soften the blow of social exclusion on themselves and on targets by choosing a form of social exclusion that includes the target in the interaction: explicit rejection. Instead of excluding targets by ignoring them (ostracism) or confusing them (ambiguous rejection), sources should opt to have a clear, explicit verbal dialog to buffer the effects of the social exclusion. Our review of disparate literatures from psychology, business, and communications also suggests several avenues that sources might pursue when constructing the language of their explicit rejection. Merely choosing an explicit rejection will not suffice: sources have to be thoughtful and sincere in their choices in everything from content to structure. Specifically, we predict that positive regard and alternatives will be associated with better goal achievement, whereas apologies will be associated with poorer goal achievement. Furthermore, we predict a Goldilocks’ principle of rejection length: rejections should not be too short or too long, but rather they should just right (i.e., be commensurate with the length of the request or the severity of the rejection). In summary, our framework suggests new directions for gaining the empirical insight needed to help sources choose wisely between the different forms of rejection.

## Author Contributions

GF and JB wrote the manuscript. KW and JB provided major revisions on the manuscript. All three authors developed the ideas in the manuscript.

## Conflict of Interest Statement

The authors declare that the research was conducted in the absence of any commercial or financial relationships that could be construed as a potential conflict of interest.

## Footnotes

1 A recent meta-analysis on the effects of social exclusion, specifically ostracism, did not find any cross-cultural differences for effects on targets’ fundamental needs (Hartgerink et al., 2015).
